# Synthesis of an Addition-Crosslinkable, Silicon-Modified Polyolefin via Reactive Extrusion Monitored by In-Line Raman Spectroscopy

**DOI:** 10.3390/polym13081246

**Published:** 2021-04-12

**Authors:** Steffen Ulitzsch, Tim Bäuerle, Mona Stefanakis, Marc Brecht, Thomas Chassé, Günter Lorenz, Andreas Kandelbauer

**Affiliations:** 1 Center for Process Analysis & Technology (PA&T), School of Applied Chemistry, Reutlingen University, Alteburgstrasse 150, 72762 Reutlingen, Germany; steffen.ulitzsch@Reutlingen-University.de (S.U.); tim.baeuerle@reutlingen-university.de (T.B.); mona.stefanakis@reutlingen-university.de (M.S.); marc.brecht@reutlingen-university.de (M.B.); guenter.lorenz@reutlingen-university.de (G.L.); 2 Reutlingen Research Institute (RRI), Reutlingen University, Alteburgstrasse 150, 72762 Reutlingen, Germany; 3 Institute of Physical and Theoretical Chemistry, University of Tübingen, Auf der Morgenstelle 18, 72076 Tübingen, Germany; Thomas.Chasse@uni-tuebingen.de

**Keywords:** reactive extrusion, silane modification, hydride modification, vinyltetramethyldi-siloxane (VTMDS), response surface analysis, ethylene-propylene copolymer (EPM), grafting, process analytical technology (PAT), in-line spectroscopy, in situ analysis

## Abstract

We present the modification of ethylene-propylene rubber (EPM) with vinyltetra-methydisiloxane (VTMDS) via reactive extrusion to create a new silicone-based material with the potential for high-performance applications in the automotive, industrial and biomedical sectors. The radical-initiated modification is achieved with a peroxide catalyst starting the grafting reaction. The preparation process of the VTMDS-grafted EPM was systematically investigated using process analytical technology (in-line Raman spectroscopy) and the statistical design of experiments (DoE). By applying an orthogonal factorial array based on a face-centered central composite experimental design, the identification, quantification and mathematical modeling of the effects of the process factors on the grafting result were undertaken. Based on response surface models, process windows were defined that yield high grafting degrees and good grafting efficiency in terms of grafting agent utilization. To control the grafting process in terms of grafting degree and grafting efficiency, the chemical changes taking place during the modification procedure in the extruder were observed in real-time using a spectroscopic in-line Raman probe which was directly inserted into the extruder. Successful grafting of the EPM was validated in the final product by ^1^H-NMR and FTIR spectroscopy.

## 1. Introduction

High-performance polymers receive considerable attention due to their special pro-perty profiles, enabling their usage in highly demanding application fields. Although standard thermoplastic materials like polyolefins are as such often insufficient, their performance can be enhanced by crosslinking them using various physical and chemical methods [1]. By grafting organosilanes onto them, an addition-crosslinkable system is produced that allows the formation of covalent crosslinkages via the grafted silane functionalities. Prominent examples include the vinyl trimethoxysilane (VTMS) grafted polyolefines ethylene propylene (EPM-g-VTMS) [2] and ethylene octene (EOC-g-VTMS) [3,4], co-polymers that combine the performance characteristics of poly-olefinic systems with silicon-containing polymers.

In the crosslinked state, high-performance synthetic elastomers are obtained that can be used, for instance, as sealants in the automotive and construction industries due to their extraordinary chemical stability, mechanical performance and durability. These properties make them also suitable as membrane materials for polymer electrolyte membrane (PEM) fuel cells [5,6,7,8,9,10]. Due to their chemical inertness and inherent biocompatibility, such elastomers may also be well suited as implant materials for biomedical applications, among many other applications [11,12,13]. Silicone rubbers are, for example, well known as breast implant materials [14].

In this work, a new addition-crosslinkable base-polymer with the potential for high-performance applications as synthetic rubber was produced and the possibility for in-line process control via in situ Raman spectroscopy was demonstrated. EPM was grafted with vinyltetramethyldisiloxane (VTMDS) via a reactive extrusion process to yield EPM-g-VTMDS. Before grafting, VTMDS was freshly prepared and subsequently purified via distillation.

The reactive extrusion process was monitored in-line by means of in situ Raman spectroscopy via a probe integrated in the extrusion line. Multivariate calibration of the Raman spectra with chemical properties was carried out based on principal component analysis (PCA) and partial least squares regression (PLS-R). With in-line Raman/PCA it was possible to quantify the amounts of grafting monomer during reactive extrusion. The grafting degree was determined using ^1^H-NMR spectroscopy. These data were used to calibrate the in-line Raman spectra to build a quantitative PLS-R model for predicting the grafting degree based on Raman signals. Thus, it is shown that both raw material consumption and formation of the grafted material can be monitored using in-line Raman spectroscopic probes directly in the process.

## 2. Materials and Methods

### 2.1. Chemicals

The liquid ethylene-propylene copolymer (EPM; Trilene^®^ CP-80) was purchased from Lion Elastomers, LLC (Geismar, Los Angeles, CA, USA). The peroxide initiator 2,5-dimethyl-2,5-di(tert-butylperoxy)hexane (DTBPH; Luperox^®^ 101) was supplied from Arkema (Colombes, France). Tetramethyldisiloxane (M1) and divinyltetramethyldisiloxane (M2) were kindly donated by CHT Germany GmbH (Tübingen, Germany). Vinyltetramethyldisiloxane (VTMDS, M3) was synthesized and purified via fractional distillation prior to the grafting reaction. Deuterated chloroform (99.8%) was purchased from Deutero GmbH (Kastellaun, Germany) and dried over 3 Å molecular sieves. Benzyl benzoate, trifluoromethanesulfonic acid and sodium hydrogen carbonate were received from Sigma-Aldrich Chemie GmbH (Taufkirchen, Germany).

### 2.2. Synthesis of Vinyltetramethyldisiloxane (VTMDS, M3)

**Figure 1:**
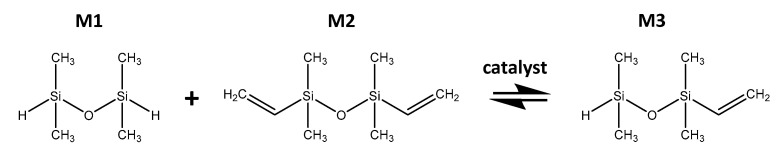
Synthesis of vinyltetramethyldisiloxane (M3; VTMDS) by equilibration of tetramethyldisiloxane (M1) and divinyltetramethyldisiloxane (M2) with trifluoromethanesulfonic acid as a catalyst.

The synthesis of vinyltetramethyldisiloxane (VTMDS, M3) was performed in a 5-L round-bottom flask. The equilibration reaction is shown in Figure 1. For the reaction, 1675.23 g tetramethyldisiloxane (M1, 134.32 g/mol) and 2324.77 g divinyltetramethyldisiloxane (M2, 186.40 g/mol) were combined in a 1:1 molar ratio, then 4.00 g acid was added and the mixture was stirred. At-line monitoring via infrared spectroscopy showed rapid equilibration within less than 1 min at a starting temperature of 25 °C without external heating. The equilibrium reaction was stopped by removing the catalyst. For this purpose, 40 g of each water and sodium hydrogen carbonate were added until the solution was neutral to slightly alkaline. The phases were separated via a separating funnel. VTMDS was purified by fractional distillation before further use.

### 2.3. Silane Grafting by Reactive Extrusion

**Figure 2:**
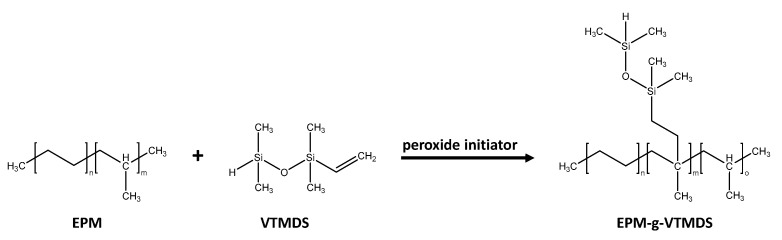
Peroxide-initiated grafting reaction of VTMDS onto ethylene-propylene rubber (EPM).

The peroxide-initiated grafting reaction of VTMDS onto EPM was done with a co-rotating twin-screw extruder (Coperion ZSK 18, Stuttgart, Germany) with a screw diameter of 18 mm and a length/diameter (L/D) ratio of 48. The chemical reaction is shown in Figure 2. 2,5-dimethyl-2,5-di(tert-butylperoxy)hexane (DTBPH) was used as the peroxide initiator.

**Figure 3:**
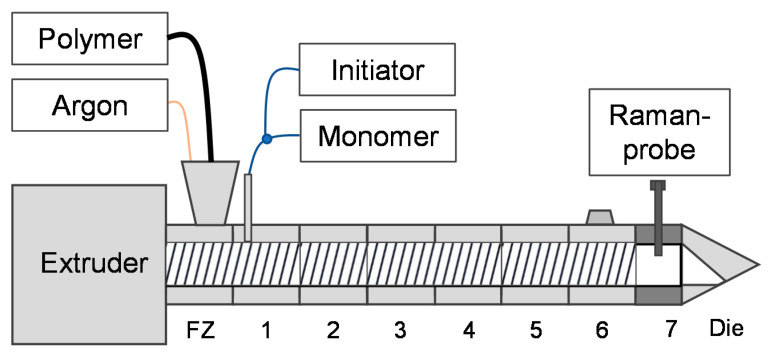
Schematic reactive extrusion setup with feeding arrangement and Raman in situ probe.

Figure 3 shows schematically the reactive extrusion setup with the feeding arrangement and Raman-probe. The liquid EPM was fed using a heated melt pump (Beinlich Pumpen GmbH, Gevelsberg, Germany). Argon was used as a protective gas and was also added at the feeding zone (FZ). At position 1, VTMDS and DTBPH were fed into the extruder via syringe pumps. Six temperature zones and the feeding zone (FZ) were heated and controlled separately. At position 7 the Raman-probe was inserted. This extruder segment was not heated.

The factor-level settings were adjusted to the factor-level combinations as required by the face-centered experimental design (FCD). The in-situ Raman spectroscopic probe placed slightly before the extruder die was used to indicate when the continuous process had stabilized after each change in factor-level settings. This guaranteed that the analyzed material samples adequately reflected the composition at a certain set of factor-level combinations. To ensure the quantitative removal of excess initiator and VTMDS residues, the material samples were placed in a vacuum-drying oven at 80 °C and dried under an argon stream for 24 h prior to further analysis.

### 2.4. Design of Experiments

A face-centered experimental design (FCD) with three varied factors was performed to determine the grafting degree and grafting efficiency. The factors studied were VTMDS feed rate (factor A), DTBPH feed rate (factor B) and relative temperature increase (factor C). The values used are listed in Table 1. The molar ratio between the peroxide initiator and grafting monomer was varied from 1:6 to 1:100. The EPM polymer feed was set at 1300 g/h: this value ensured the best possible extruder loading and feeding. All factor settings were selected to obtain the feasible limits of reactive extrusion and were determined by preliminary experiments.

The detailed settings for the heating segments in the extruder, depending on the respective FCD settings, are given in Table 2.

For planning and analyzing the experiments, the computer program Design Expert (Version: 11.1.2.0 and 12.0.12.0, Stat-Ease, Inc., Minneapolis, MN, USA) was used. To determine the relevant factors and factor interactions, an analysis of variance (ANOVA) was calculated. Only statistically significant effect terms were used to build the response surface models. As a criterion for statistical significance, a significance level of 5% (*p*-value of <0.05) was used.

### 2.5. Multivariate Data Analysis

Multivariate data analysis (MVA) was performed with “The Unscrambler X 10.5” software (Camo Analytics, Oslo, Norway). All in-line Raman spectra were pre-processed using the Savitzky–Golay 1st (smoothed) derivative (symmetric 21 points, 2nd polynomial order).

The principal component analysis (PCA) used to determine the proportion of the VTMDS feed was calculated with mean centering, cross validation (20 random segments) and the Nonlinear Iterative Partial Least Squares (NIPALS)-algorithm. Model outliers were identified in the influence plot of Hotelling’s T² values versus F-residuals (outlier limits 5% each). The wavenumbers ranged from 1800–300 cm^−1^ obtained with all 19 sample sets was used for the model.

Partial least squares regression (PLS-R) for prediction of the in-line grafting degree was performed for four factors using mean centering, cross validation (random 20 segments) and the kernel algorithm. All 19 sample sets were divided into calibration set samples (sets number 1–8, 10, 12–19) to develop the PLS-R model and the external validation samples (sets number 9 and 11) to validate the PLS-R model; here, the spectral range 900–300 cm^−1^ was used.

### 2.6. Differential Scanning Calorimetry (DSC) for Boiling Point Determination

All DSC measurements were performed on a DSC 204 F1 Phoenix from Netzsch (Selb, Germany). Thirteen- to fifteen-milligram samples were weighed into aluminum DSC crucibles (Concavus^®^, Netzsch, Selb, Germany) and covered with a pierced lid. The temperature interval ranged from −10 °C to 200 °C; the heating rate was 15.0 K/min. The measurements were performed with a temperature and heat flow calibration. Nitrogen was used as a purge gas with a gas flow of 40 mL/min and as a protective gas with a gas flow of 60 mL/min. Data analysis was done using Netzsch Proteus-Thermal Analysis-software version 7.1.0.

### 2.7. Attenuated Total Reflection Fourier Transform Infrared Spectroscopy (ATR-FTIR) for Distillation Monitoring

Attenuated total reflection Fourier transform infrared spectroscopy (ATR-FTIR) was used for monitoring the distillation (PerkinElmer Inc., Waltham, MA, USA). Spectra were collected in the wavelength range from 4000–600 cm^−1^. For each single spectrum, 16 scans were accumulated.

### 2.8. Raman Spectroscopy for In-Line Monitoring, Process Control and Off-Line Spectra

A modified Alpha300 SR (WITec GmbH, Ulm, Germany) as described in [14] was used for the Raman spectroscopic off-line measurements using a 20× objective (Zeiss, EC Epiplan 20×/0.4 M27); excitation was at 532 nm. The spectra were acquired with integration time 1 s and 10 accumulations.

An extruder-compatible immersion probe with a flat sapphire window (Dynisco Inc., Franklin, MA 02038, USA) connected to a spectrometer (RXN1, Kaiser Optical Systems, Ann Arbor, MI 48104, USA) was used for the in-line Raman measurements. The spectra were acquired with an exposure time of 10 s, two accumulations and a cosmic ray removal unit.

### 2.9. Nuclear Magnetic Resonance Spectroscopy

The equilibration synthesis and grafting products were characterized by proton nuclear magnetic resonance spectroscopy (^1^H-NMR) measurements. Approximately 25 mg of sample and an additional reference for determination of the grafting degree were weighed into a sampling tube and dissolved in 1.0 mL deuterated chloroform (CDCl_3_). The measurements were performed on a Bruker AvanceTM III spectrometer (Bruker BioSpin GmbH, Rheinstetten, Karlsruhe, Germany) with a resonance frequency of 400.13 MHz, an acquisition time of 4.089 s, a relaxation delay between pulses of 1 s, a pulse width of 14 µs and a temperature of 298 K.

The NMR spectra processing and analysis was done with the software package MestreNova (Version: 14.0.1-23559, Mestrelab Research, Santiago de Compostela, Spain). Phase correction and baseline correction were performed and all spectra were referenced to CDCl_3_ at 7.26 ppm.

### 2.10. Calculation of Grafting Degree and Grafting Efficiency

Determination of grafting degree and grafting efficiency was performed in an analogous way as described in previous studies on grafting silane coupling agents onto ethylene propylene (EPM) and ethylene-octene copolymer (EOC) polyolefins [2,4]. The grafting degree as a weight percentage (wt%) was calculated according to Equation (1) [2]. Integration areas (I) of reference proton shifts of the reference standards (R) at 5.37 ppm and the grafting product (S) at 4.69 ppm were used. N is the number of protons of the hydride group, m the weighed mass, M the molar mass and P the purity of the benzyl benzoate reference standard [2].

(1) $$\mathrm{VTMDS}\;\mathrm{grafting}\;\mathrm{degree}\;(\mathrm{wt}\%)=\frac{I(S)}{I(R)}\times \;\frac{N\left(R\right)}{N\left(S\right)}\;\times \;\frac{m\left(S\right)}{m\left(R\right)}\;\times \frac{M\left(S\right)}{M\left(R\right)}\;\times \;P\left(R\right)\times \;100\%$$

Grafting efficiency in wt% was calculated using Equation (2). For this, the measured VTMDS grafting degree was divided by the grafting degree theoretically expected under the assumption of 100% conversion [2,4].

(2) $$\mathrm{VTMDS}\;\mathrm{grafting}\;\mathrm{efficiency}\;\left(\%\right)=\;\frac{\mathrm{measured}\;\mathrm{grafting}\;\mathrm{degree}\;\mathrm{VTMDS}}{\mathrm{theoretical}\;\mathrm{grafting}\;\mathrm{degree}\;\mathrm{VTMDS}}\;\times 100\%$$

## 3. Results and Discussion

### 3.1. Vinyltetramethyldisiloxane (VTMDS) as Grafting Monomer for Reactive Extrusion

**Figure 4:**
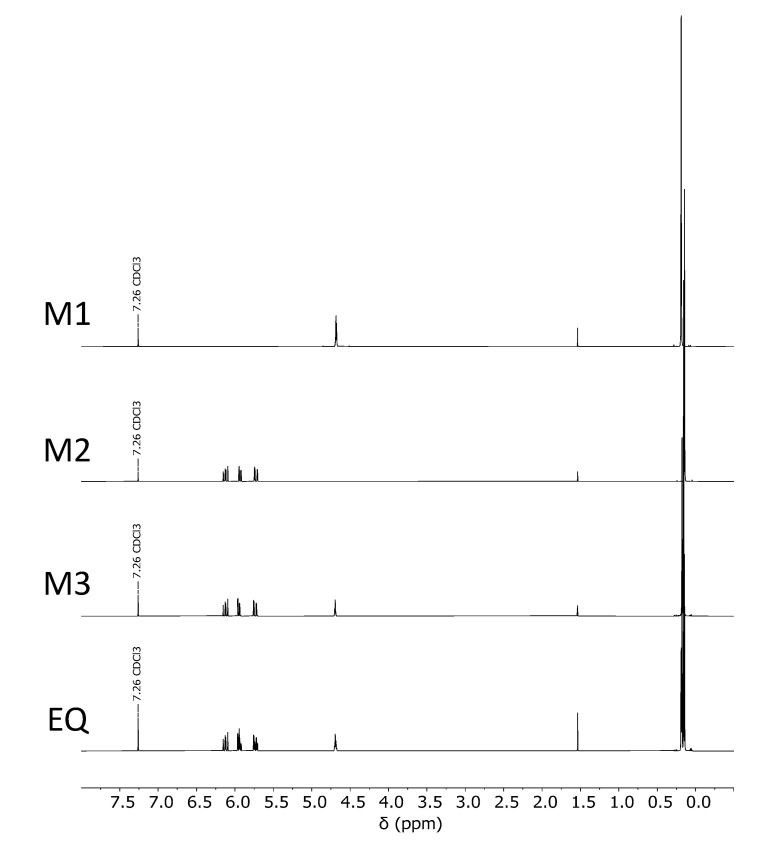
^1^H-NMR spectra of tetramethyldisiloxane (M1), divinyltetramethyldisiloxane (M2), vinyltetramethyldisiloxane (M3; VTMDS) and the equilibration reaction (EQ).

The ^1^H-NMR spectra of the equilibration reaction are shown in Figure 4. Tetramethyldisiloxane (M1), divinyltetramethyldisiloxane (M2) and vinyltetramethyldisiloxane (M3; VTMDS) are the pure substances and EQ is the unpurified reaction product. In EQ, all single substances are present in a molar ratio of 1:1:1. At 7.26 ppm the CDCl_3_ peak of the solvent can be observed, and at 1.56 ppm the water peak of the water which diffused into the sample due to air humidity can be seen. The SiCH_3_ methyl groups are in the range of 0–0.35 ppm, the SiH hydride group at 4.5–4.9 ppm and the vinyl groups in the range of 5.55–6.3 ppm.

**Figure 5:**
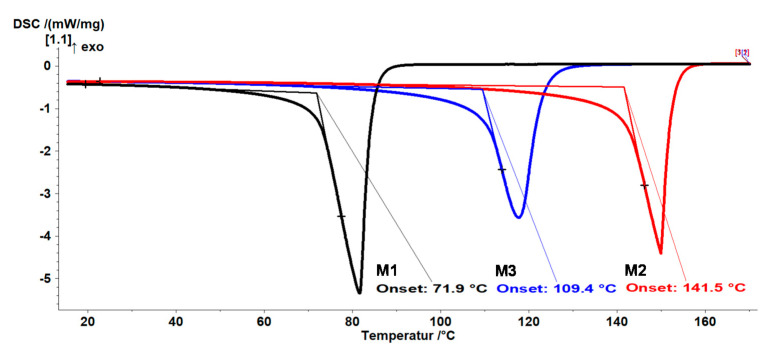
Differential scanning calorimetry (DSC) traces of tetramethyldisiloxane (M1), vinyltetramethyldisiloxane (M3; VTMDS) and divinyltetramethyldisiloxane (M2) used to determine the boiling points for distillation. A heating rate of 15.0 K/min was used and nitrogen was used as purge gas with a gas flow of 40 mL/min and as protective gas with a gas flow of 60 mL/min.

To be able to separate the substances by means of fractional distillation, the boiling points of all three compounds were determined using differential scanning calorimetry (DSC). For this purpose, DSC curves of the three pure substances were measured and the boiling points were determined over the onset, as shown in Figure 5. The following boiling points were determined: M1 = 72 °C, M2 = 142 °C and M3 = 109 °C. The boiling point determination for M3 was the most relevant as there were no standardized values available in the scientific literature for this. Due to the boiling point differences, distillation was performed without a vacuum.

**Figure 6:**
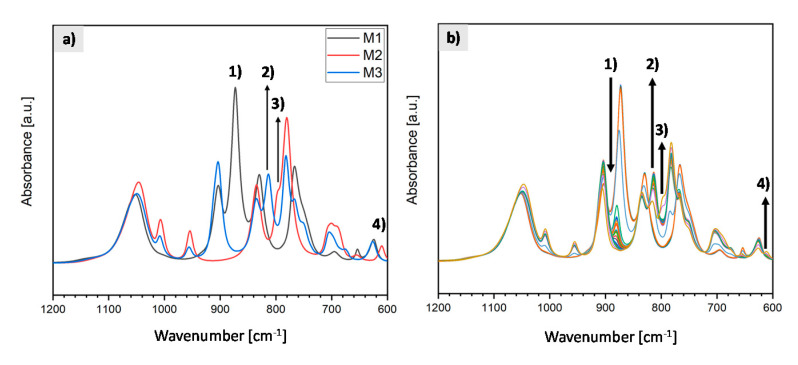
Attenuated total reflection (ATR)-FTIR spectra of (**a**) tetramethyldisiloxane (M1), divinyltetramethyldisiloxane (M2) and vinyltetramethyldisiloxane (M3; VTMDS) and (**b**) the distillation process of vinyltetramethyldisiloxane (M3; VTMDS). (1) M1 decreases and upon its complete removal separation of M3 took place. (2) M3 increases. (3) and (4) M2 increases.

At-line monitoring via ATR, FTIR was performed during distillation to achieve the maximum yield and purity of VTMDS (M3). Figure 6a shows the infrared spectra from 1200 cm^−1^ to 600 cm^−1^ of the pure substances tetramethyldisiloxane (M1), divinyltetramethyldisiloxane (M2) and vinyltetramethyldisiloxane (M3; VTMDS). Figure 6b shows a time series of infrared spectra from the distillation process. M1 is mainly characterized by medium to strong Si-H deformation vibrations at 903 cm^−1^ and 837 cm^−1^. At the beginning of the distillation, the 837 cm^−1^ vibration, denoted by (1), decreases (Figure 6b). The increase of band (2) at 814 cm^−1^ in the spectrum describes the increase in the Si-H group in the product M3, determined by the Si-H deformation vibration. When band (1) was minimal and band (2) was maximal, the extraction of M3 was started [15,16].

The vibrations at 1007 cm^−1^ (trans CH wagging) and 954 cm^−1^ (CH_2_ wagging) are associated with the vinyl group Si–CH=CH_2_ of the reactant M2. At the end of the distillation, the increase of shoulder (3) and the increase of band (4) are crucial for M2 (Figure 6b). As soon as bands (3) and (4) increase and, thus, more M2 is present again, pure M3 is no longer present and the distillation is ended. Shoulder (3) at 797 cm^−1^ is caused by the medium to strong Si–CH_3_ or Si-C rocking vibrations. Band (4) at 610 cm^−1^ describes the broad symmetric Si-O-Si stretching band for disiloxanes. Both bands are present only in M2 at these wavenumbers (see Figure 6a). Minor shifts in the bands during the reaction and distillation process are caused by changes in the overall electric dipole moment of the molecules and their interaction with the surrounding molecules. Due to the progressive reaction and the formation of M3, which contains both Si-H and Si–CH=CH_2_ as functional groups, the net electric dipole moment of M3 changes, despite the stoichiometric conservation of the Si-H and Si–CH=CH_2_ functional groups. As a result, in the basic structure of the molecule M3, the Si–CH_3_ and Si-O-Si vibrations are also affected, and these bands are slightly shifted. Due to the decrease in the concentration of M3 during the course of the reaction and the resulting change in molecular composition, the direction of this shift is reversed [15,16]. VTMDS (M3) was further used in the grafting reactions.

### 3.2. Structural Characterization of the Reaction Products

**Figure 7:**
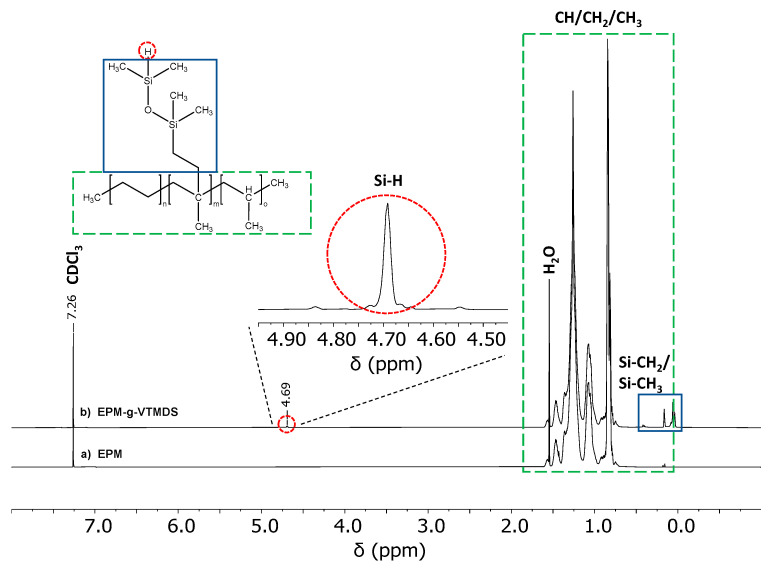
^1^H-NMR spectra of (**a**) raw unmodified EPM and (**b**) EPM-g-VTMDS.

The successful grafting reaction of VTMDS onto EPM, as monitored by ^1^H-NMR, is shown in Figure 7. Figure 7a shows the EPM polymer. Figure 7b shows the grafted product, EPM-g-VTMDS. The relevant ranges are 0–1.8 ppm for the mainly polyolefinic CH/CH_2_/CH_3_ groups and −0.25–0.5 ppm for the Si–CH_2_/Si–CH_3_ groups. The signal at a chemical shift of 4.69 ppm is characteristic for the SiH hydride group and indicates successful hydride modification of the polyolefin.

### 3.3. Quantitative Determination of Grafting Degree and Grafting Efficiency

The results of the experimental design are summarized in Table 3. The grafting degree was calculated with Equation (1) and the grafting efficiency was calculated using Equation (2). All experiments were performed in one block. The experiments are listed in the actual run order. To determine the experimental error with high accuracy, six center point (CP) experiments at intermediate factor-level settings were performed.

For all experiments the overall range of grafting degrees achieved was between 0.33 wt% and 1.92 wt%. The grafting efficiency for all experiments was between 16.14% and 85.03%. This is well within the expected range of grafting degrees observed with similar grafting reactions performed via reactive extrusion in earlier studies [2,4]. All experiments were included in the model building.

### 3.4. Effect of Process Variables on Grafting Degree

To determine the effect of the varied process parameters on the grafting degree, ANOVA was performed (Table 4). Six effect terms with a *p*-value less than 0.05 were obtained to build a response surface model containing only statistically significant model terms. The various coefficients of determination calculated for model evaluation indicate that the model fits the data very well (R^2^ = 0.9690), contains an appropriate number of model terms (R^2^_adjusted_ = 0.9546) and allows good and robust predictions (R^2^_predicted_ = 0.9114).

The linear contributions of the factor effects VTMDS feed rate (factor A) and DTBPH feed rate (factor B) on the grafting degree are the most important effects that mainly determine the system behavior. They are both in the same order of magnitude and about three times higher than the linear impact of temperature increase (factor C). In addition, changes in the DTBPH feed rate result in non-linear response behavior of the grafting degree, as indicated by the significant non-linear effect term B^2^. The positive values indicate that an increase in factor level leads to a higher grafting degree. VTMDS and DTBPH feed rates are involved in a second-order interaction (the two-factor interaction term AB). This means that the effects of changes in VTMDS feed rate depend on the settings of DTBPH feed rate at which the changes in VTMDS feed rate are made, or, in other words, that VTMDS and DTBPH feed rates must be adjusted together in a coordinated manner in order to achieve a desired grafting degree. The temperature increase also induces a non-linear response of the grafting degree. This shows that there is an optimum value for DTBPH feed rate and temperature increase, whereas an increase in VTMDS feed rate always leads to higher grafting degrees. The model equation in terms of coded values summarizes the relative importance of factor effect terms: $\mathrm{grafting}\;\mathrm{degree}\;\mathrm{VTMDS}\;\left(\mathrm{wt}\%\right)=1.24+0.3260\times A+0.3290\times B+0.1290\times C+0.1475\times \mathrm{AB}-0.1875\times B^{2}-0.1675\times C^{2}$.

The molar ratio between VTMDS monomer and peroxide has no effect on the grafting degree. Specific values for the target response grafting degree can be calculated using the factor effects equation in actual terms: $\mathrm{grafting}\;\mathrm{degree}\;\mathrm{VTMDS}\;\left(\mathrm{wt}\%\right)=-0.137224+1.15286\times A+81.38776\times B+0.023200\times C+210.71429\times \mathrm{AB}-3826.53061\times B^{2}-0.000419\times C^{2}$.

**Figure 8:**
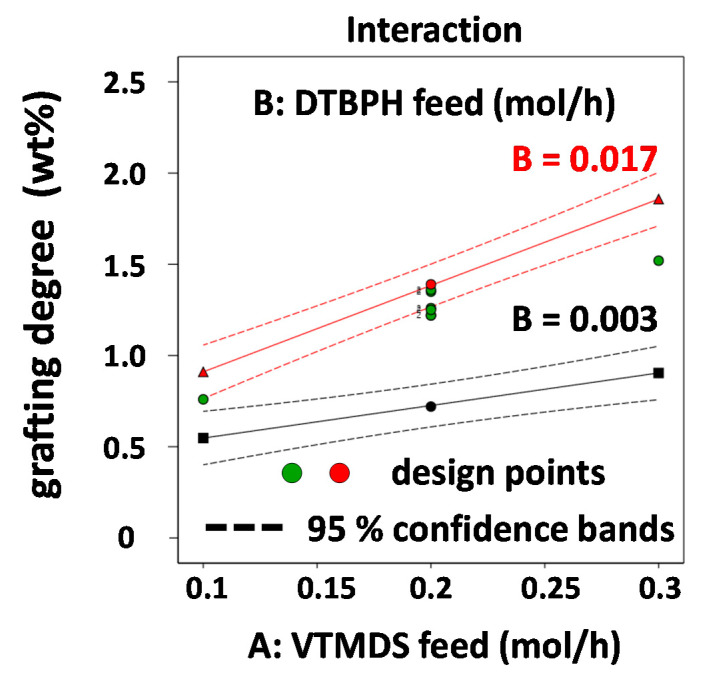
Interaction plot for the two-factor interactions between VTMDS and DTBPH feed rates (AB) on the grafting degree response.

Figure 8 depicts an interaction plot illustrating the second order interaction between the VTMDS feed rate and the DTBPH feed rate (Figure 8). This is the only interaction affecting the grafting degree in this grafting reaction. The obvious effect of this synergism is that the positive effect of increasing VTMDS feed rate on the grafting degree is further enhanced by using higher feed rates of peroxide initiator, suggesting that overall a higher number of radicals is generated, initiating the grafting reaction.

**Figure 9:**
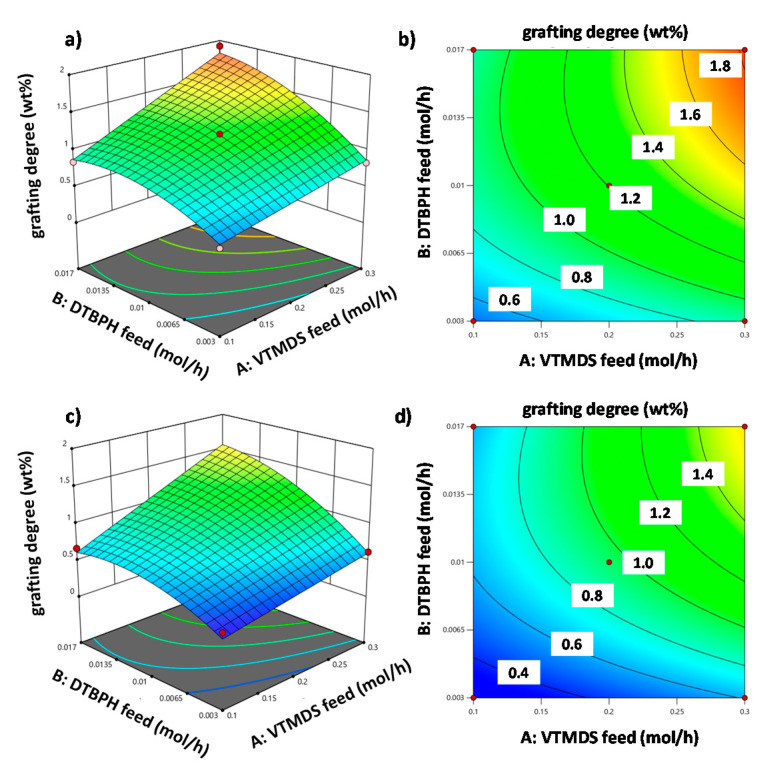
Grafting degree as a function of the VTMDS and DTBPH feed rates for two scenarios: (1) 3D response surface plot (**a**) and corresponding contour line plot (**b**) at the high level for the factor “temperature increase”, and (2) 3D response surface plot (**c**) and corresponding contour line plot (**d**) at the low level for the factor “temperature increase”.

The effects of the two most important process factors on the grafting degree are visualized in Figure 9 as 3D response surface and contour line plots. Figure 9a,b shows the effects of VTMDS and DTBPH feed rates at the highest value used for the temperature increase, whereas Figure 9c,d shows these effects at the lowest value for the temperature increase.

The effects of process factor variations (i.e., the coefficients in the factor effects equation in terms of coded values) on grafting degree are in good agreement with the factor effects obtained in earlier studies of similar systems, in which the grafting reactions of vinyltrimethoxysilane (VTMS) onto ethylene-propylene (EPM) [2] and ethylene-octene copolymer (EOC) [4] via reactive extrusion were investigated using response surface methodology. The same overall trends were found in all three systems. All three reactive extrusion processes are governed by non-linear factor effects and the same types of second-order interaction effects. For instance, the linear components of the temperature effect on the degree of grafting were in a comparable order of magnitude in all systems: EPM-g-VTMS: 0.10 [2]; EOC-g-VTMS: 0.10 [4]; EPM-g-VTMDS: 0.13 (this work), although in [4] the temperature was varied within a slightly narrower range from 100 °C–220 °C instead of 80 °C to 220 °C. Although most effects were similar, some notable differences should be pointed out. While the linear factor effect of variations in the silane monomer feed rate on the grafting degree in the EPM-g-VTMDS system studied in the present work (0.33) is practically the same as that for the EPM-g-VTMS system (0.35), [2], it is only about 25% of the factor effect found for the EOC-g-VTMS system in Ulitzsch et al. [4] (1.17). Obviously, in the ethylene-octene copolymer (EOC) H is abstracted more readily and this seems to have a positive effect on the obtainable grafting degrees. The linear effect term for the peroxide feed rate, as another interesting difference, was of comparable magnitude in the EPM-g-VTMS (0.60) [2] and EOC-g-VTMS (0.66) [4] systems but is almost twice as large as the effect found for the EPM-g-VTMDS system (0.33) in the present study. This difference can be attributed to the nature of the grafting monomer VTMDS. Unlike VTMDS, VTMS carries three methoxy groups that exhibit a positive electron-inducing effect. This makes the double bond of VTMS more reactive. These differences illustrate the importance of considering the nature of the polymer backbone (type of polyolefin) and the nature of the grafting monomer (silane coupling reagent) when designing the grafting process. In all three systems, the interaction between the grafting monomer and peroxide initiator feed rates (AB) was highly significant [2,4].

### 3.5. Effect of Process Variables on Grafting Efficiency

To predict grafting efficiency, response surface analysis yielded a model containing seven relevant factor effect terms. Again, only terms with a *p*−value less than 0.05 were included. The ANOVA is presented in Table 5. With coefficients of determination R^2^ = 0.9772, R^2^_adjusted_ = 0.9638 and R^2^_predicted_ = 0.9206, the model displayed good experimental fit, was not overfitted and yielded robust predictions.

Grafting efficiency depends non-linearly on VTMS and DTBTH feed rates. These two factors are also the most important effects that mainly determine the system’s behavior. They are both in the same order of magnitude. Increasing the VTMDS feed rate has a negative effect on grafting efficiency, whereas grafting efficiency is improved when the DTBPH feed rate is increased. Again, VTMDS and DTBPH feed rates are involved in a second-order interaction. Grafting efficiency also depends non-linearly on the temperature increase, although its overall effect is slightly smaller than the effects of the feed rates. The relative magnitudes of the significant effects are summarized in the factor effects equation in terms of coded factors: $\mathrm{grafting}\;\mathrm{efficiency}\;\mathrm{VTMDS}\;\left(\%\right)=51.14-14.52\times A+15.03\times B+5.99\times C-3.01\times \mathrm{AB}+8.03\times A^{2}-7.71\times B^{2}-6.93\times C^{2}$.

The molar ratio between silane grafting agent and peroxide initiator had no effect on the grafting efficiency. Specific values for the grafting efficiency can be calculated from the factor effects equation in terms of actual values: $\mathrm{grafting}\;\mathrm{efficiency}\;\mathrm{VTMDS}\;\left(\%\right)=+53.56777-423.37234\times A+6154.19481\times B+0.992823\times C-4292.85714\times \mathrm{AB}+802.72727\times A^{2}-157403\times B^{2}-0.017332\times C^{2}$.

**Figure 10:**
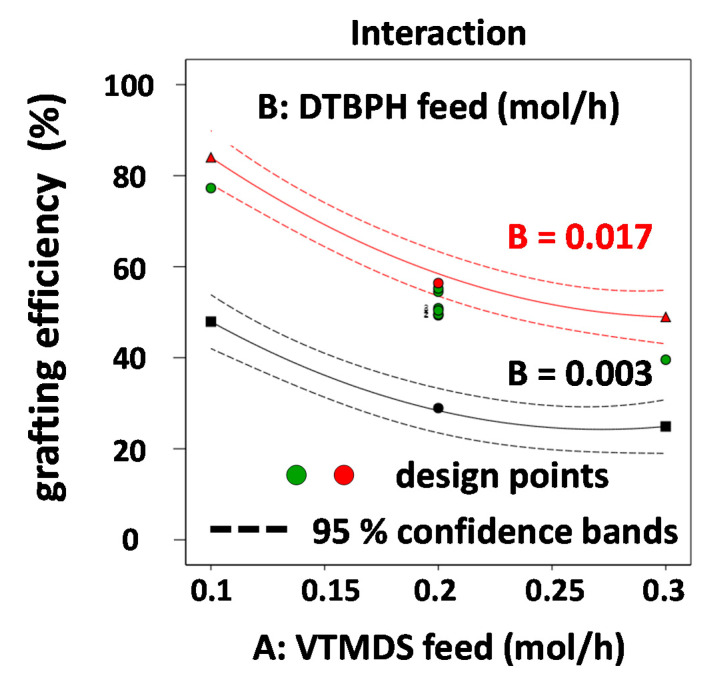
Interaction plot for the 2 FIAs of VTMDS and DTBPH feed rates (AB) on the grafting efficiency response.

The interaction between VTMDS and DTBPH feed rates is visualized in the interaction plot presented in Figure 10.

**Figure 11:**
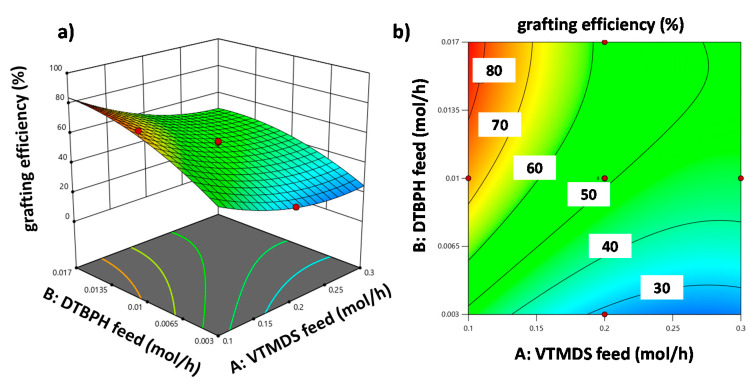
(**a**) 3D response surface plot and (**b**) contour plot for the response grafting efficiency showing the dependence on VTMDS and DTBPH feed rates at intermediate factor level setting for the temperature increase.

The effects of the process factors on grafting efficiency are illustrated in Figure 11. The non-linear effects of all three factors and the second-order interaction effect cause the response surface to have the form of a twisted saddle, which is shown in the 3D response surface plot in Figure 11a. The contour line plot shown in Figure 11b enables quantitative conclusions to be drawn by projecting the response surface in two dimensions. In Figure 11, a scenario is depicted in which the level of the factor ”temperature increase” is set to an intermediate level (the center point settings).

As with the grafting degree, the factor effects on grafting efficiency (i.e., the coefficients of the factor effects equation in terms of coded factors) are also in good agreement with effects found earlier with the EPM-g-VTMS [2] and EOC-g-VTMS [4] systems. Again, the linear factor effect of peroxide feed rate is comparable in all three systems, with EPM-g-VTMS: 17.8 [2], EOC-g-VTMS: 14.4 [4] and EPM-g-VTMDS: 15.0 (this work). The effect of silane monomer feed rate, however, although in the same order of magnitude with the VTMS systems (−10.0 [2] and −9.6) [4], was about 1.5 times larger in the EPM-g-VTMDS system (this work). Since in [2] and [4] VTMS was used as the grafting reagent, this difference is attributed to the chemical nature of VTMDS.

The effect of temperature increases noticeably in the series EOC-g-VTMS [4] < EPM-g-VTMS, [2] < EPM-g-VTMDS. In [2], liquid EPM was used, whereas in [4], EOC was applied as a solid granulate. Since in the present study and [2] the backbone polymer was the same, the additional positive effect on grafting efficiency observed is due to the different grafting monomer. Thus, VTMDS is more favorable for obtaining higher grafting efficiency than VTMS [2,4].

### 3.6. Process Window for the Grafting Reaction at Reactive Extrusion

Both models, the one for the grafting degree and the one for the grafting efficiency are sufficiently good and can be used to define a process window which accounts for a good compromise between grafting degree and grafting efficiency. The hydride content of silicones is often given in terms of molality (mmol/g) in order to facilitate the dosage of appropriate molar ratios of the vinyl and hydride components for the hydrosilylation reaction. To obtain the hydride content in wt% the grafting degree is divided by 160 (M(H) = 1 g/mol; M(VTMDS) = 160 g/mol). $\mathrm{hydride}\;\mathrm{content}\;(\mathrm{wt}\%)=\frac{M\left(H\right)}{M\left(\mathrm{VTMDS}\right)}\cdot \mathrm{grafting}\;\mathrm{degree}\;(\mathrm{wt}\%)$. The molality is calculated by $\mathrm{molality}\;(\mathrm{mmol}/g)=\frac{\mathrm{hydride}\;\mathrm{content}\;(\mathrm{wt}\%)}{M(H)}\times \frac{1\;}{100\%}\times \frac{1000\;\mathrm{mmol}}{1\;\mathrm{mol}}$. Optimization of the reactive extrusion process is targeted at 0.1 mmol hydride/g. This amount is favorable for subsequent crosslinking of the hydrosilylated polyolefin. This molality of 0.1 mmol hydride/g corresponds to a grafting degree of 1.6%.

**Figure 12:**
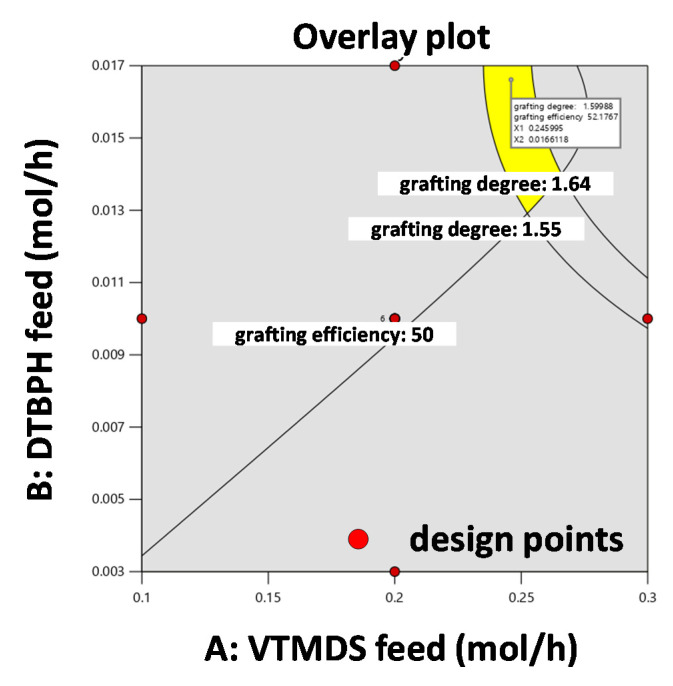
Overlay plot for the process window of 1.55 wt%–1.64 wt% grafting degree and grafting efficiency of >50% with the target of 1.6 wt% grafting degree with the correlation of VTMDS feed and DTBPH feed and all other factors at intermediate factor-level settings.

Figure 12 shows the overlay plot defining the process window to obtain a grafting degree 1.55 wt%–1.64 wt% at a grafting efficiency > 50%. In this optimization scenario, reactive extrusion is run at the intermediate setting for the temperature increase (i.e., the CP setting). This corresponds to a temperature range from 100 °C to 200 °C. The area colored in yellow highlights all possible combinations of VTMDS and DTBPH feed rates at this temperature increase that yield satisfactory grafting degrees and grafting efficiencies.

**Figure 13:**
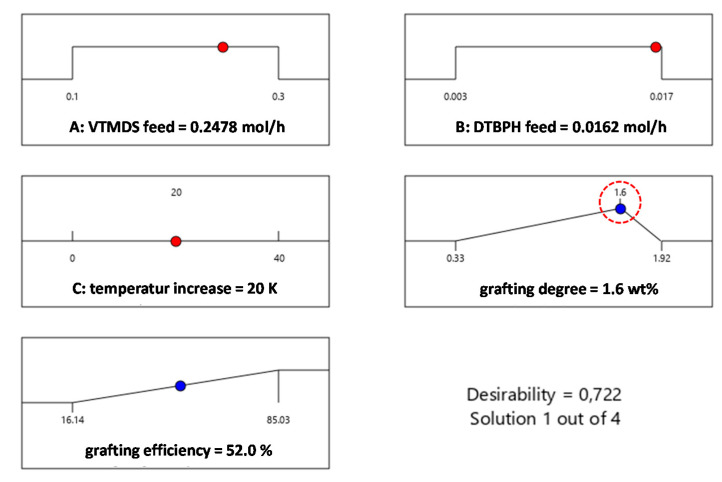
Process optimization for a grafting degree of 1.6 wt% and high grafting efficiency.

The process parameters calculated for the validation experiment are shown in Figure 13. The selected combination was calculated to achieve a molality of 0.1 mmol hydride/g, which corresponds to a grafting efficiency of 1.6 wt% VTMDS. The grafting efficiency here was expected to be maximized and was predicted to be 52%. The temperature increase covered a temperature range of 100 °C–200 °C. The VTMDS feed was set to 0.2478 mol/h and the DTBPH feed was set to 0.0162 mol/h.

The validation experiment resulted in a grafting degree of 1.64 wt%, corresponding to a molality of 0.10 mmol hydride/g. The grafting efficiency was also within the target range, at 53.17%, indicating that the models for both target responses are valid and lead to reliable predictions.

### 3.7. Data Selection and Pre-Processing for Multivariate Analysis

In this work, in-line Raman spectroscopy was combined with multivariate data analysis for the characterization of the process to determine the proportions of VTMDS and to predict the grafting degree of the final product. A total of 20 trials were run in a rando-mized trial sequence. During each experimental run, Raman spectra were continuously recorded in-line every 30 s, to monitor the entire process. All spectra distorted by measuring artefacts, caused for instance by gas bubbles, were eliminated from the data set. The dataset contained only spectra obtained under stable process conditions after adjusting the pre-defined process factor-level settings.

Principal component analysis (PCA) with 188 spectra from all 19 experiments was used to quantify the VTMDS concentration. Spectra from 17 experiments were used for the calibration model for the PLS-R of the grafting degree. For a low and a high grafting degree, two randomly selected experiments were taken for external validation. The details of the respective experiments and models can be found in Table 6.

**Figure 14:**
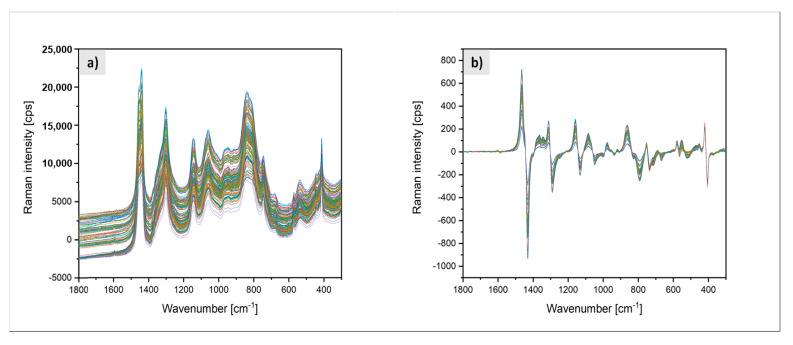
(**a**) Time series of in-line Raman spectra taken during the reactive extrusion process, (**b**) spectra after pre-processing with spectral pre-processing by the Savitzky−Golay 1st (smoothed) derivative (symmetric 21 points, 2nd polynomial order).

All Raman spectra were preprocessed using the Savitzky−Golay 1st derivative (smoothed, symmetric 21 points, 2nd polynomial order). They are shown in Figure 14.

**Figure 15:**
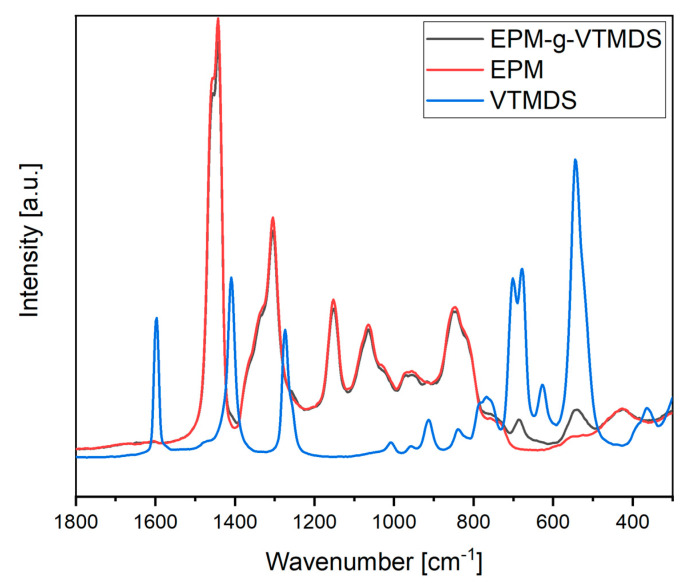
Off-line Raman spectra of VTMDS, EPM and EPM-g-VTMDS.

To interpret the in-line Raman spectra, which contained superimposed information of the entire process, they were compared with off-line Raman spectra obtained from the pure substances of the reactants (EPM and VTMDS) and the product EPM-g-VTMDS (Figure 15). The in-line process spectra contain information not only on the mixture of the three pure substances, but also on the initiator present in traces and other potential by-products. They also reflect the effects of all variations in process parameter settings introduced during the factorial experiment. Shifts in the bands are possible while comparing the in-line and off-line spectra since the surrounding matrix has an influence on the polarizability of the electron shell of the molecule and thus on the position of the Raman bands. In addition, different Raman instruments were used for recording the in-line and the off-line spectra. Therefore, spectral ranges of Raman oscillations instead of specific single values for the Raman vibration bands are given for the relevant signals in the in-line spectra.

The predominant information visible in the in-line Raman data is very similar to that of pure EPM. Since EPM forms the backbone for the grafted polymer and thus constitutes the bulk of the reactant, this is reflected by the spectral signature of the process. Raman active bands in EPM are mainly C−C−C, −CH_2_− or −CH_3_− vibrations. For example, the C−C−C vibrations for aliphatic and branched polymers occur from 1100–1040 cm^−1^, at 970 cm^−1^, from 900–800 cm^−1^, from 540–485 cm^−1^ and at 300 cm^−1^. Between 1305 cm^−1^ and 1295 cm^−1^ is the medium-strength −(CH_2_)_n_− deformation vibration, and at 735 cm^−1^ the −(CH_2_)_3_− rocking vibration. In the spectral range between 560 cm^−1^ and 420 cm^−1^ the CH_2_ wagging, C−CH_3_ stretching and CH_2_ rocking vibrations are found with different intensities. For the monomer VTMDS, the Si–CH=CH_2_ vibrations from 1615–1590 cm^−1^ (C=C stretching), from 1410–1390 cm^−1^ (CH_2_ in plan deformation), 1020–1000 cm^−1^ (trans CH wagging) and from 980–940 cm^−1^ (CH_2_ wagging) are Raman-active. Moreover, between 985–800 cm^−1^ lies the Si−H wagging deformation. Furthermore, between 770 cm^−1^ and 675 cm^−1^ are different Si−C vibrations. The symmetric Si−O−Si vibration is most pronounced between 625–480 cm^−1^ in the off-line spectra. However, since the monomer is a much smaller molecule relative to EPM, these vibrations are strongly superimposed by other signals in the in-line process spectra and are not clearly discernible. The same applies to the subtle differences between EPM and the EPM-g-VTMDS product. The spectral differences are only weakly pronounced even in the off−line spectra of the pure substances. At 763 cm^−1^, an additional shoulder can be seen in the grafted product that is not present in the EPM starting material. The strongly Raman-active Si−C stretching band is located in this region. Additional signals can also be seen at 687 cm^−1^ and at 540 cm^−1^. The signal at 687 cm^−1^ is assigned to the Si−C stretching vibration. Between 625 cm^−1^ and 480 cm^−1^ the very strong symmetric Si−O−Si stretching vibration occurs. Minimal decreases in the signal intensities are also seen in EPM-g-VTMDS. These are observed at 1454 cm^−1^, 1442 cm^−1^, 846 cm^−1^ and 820 cm^−1^. The signals between 1456–1440 cm^−1^ can be assigned to asymmetric −CH_3_ vibrations of aliphatic polymers. The changes at 846 cm^−1^ and 820 cm^−1^ are caused by changes in the polarizability of the electron shell of the C−C−C backbone. Further details regarding band assignment and the location of the bands found in the off-line Raman spectra are given in Table 7.

The use of an in situ real-time process analyzer for monitoring the grafting degree was investigated. In order to extract and interpret the superimposed and relevant information, multivariate methods PCA and PLS-R were applied.

### 3.8. Determination of VTMDS Content Using PCA

**Figure 16:**
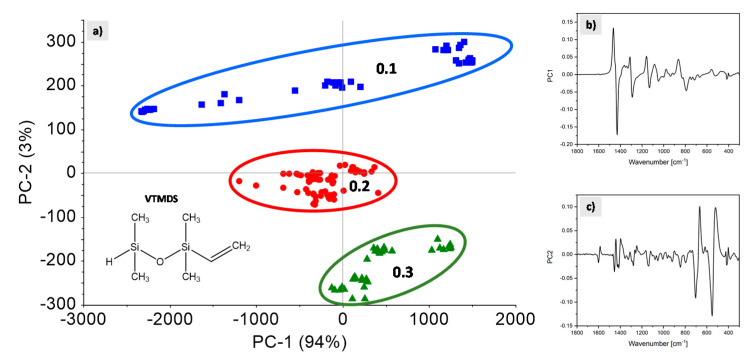
(**a**) Scatter plot for PC1 and PC2. Loadings for (**b**) PC1 and (**c**) PC2.

PCA was used to restructure the preprocessed spectroscopic data of all 19 sample sets along the maximum variance. The range from 1800 cm^−1^ to 300 cm^−1^ was investigated. The model based on two principal components explained the data variance sufficiently well (at 97%) and was used for modeling the VTMDS feed. The model was verified by random cross validation with 20 segments. Each dot shown in the graph in Figure 16a represents a Raman spectrum. The closer the dots are in the scores plot (Figure 16a), the more similar they are with respect to the PCs concerned. Conversely, samples that are more distant from each other are spectroscopically more different. The plot can be used to interpret differences and similarities among spectra. Together with the corresponding loadings plots (Figure 16b–c) for the same two components it is possible to determine which variables in the data set structured by the DoE plan are responsible for differences between sample sets.

In PC1, no clear sample grouping was evident. Possibly, the maximum variance in the data indicated the differences in the process settings and thus in the individual experiments as a whole. Temperature, the amount of initiator used (peroxide content) and the monomer feed of VTMDS were varied. Thus, the polymer was grafted to varying grafting degrees. This sum of differences in the sample sets was expressed by PC1. Furthermore, in the corresponding loadings for PC1 (Figure 16b), for example, the bands in the range 1460–1440 cm^−1^ (−CH_3_), 1310–1290 cm^−1^ (−(CH_2_)_n_−) and 1160–1130 cm^−1^ (C−C−C), indicate changes in the polarizability of the electron shell of the molecule and its surrounding matrix.

In contrast, distinct sample groupings were identified in PC2. Along the PC2 axis, clustering of the Raman spectra with respect to the applied variations in VTMDS feed rate is visible. The different experimental settings are highlighted in Figure 16a with different colors: 0.1 mol/h (blue), 0.2 mol/h (red) and 0.3 mol/h (green). This suggests that changes visible in PC2 can be attributed to variations in VTMDS feed rates. In the loadings plot for PC2 (Figure 16c) those bands where the largest changes upon variations in VTMDS feed rate occur can be identified and interpreted. The loadings at around 1410 cm^−1^ are assigned to the CH_2_ in plane deformation vibrations of the Si–CH=CH_2_ group. The loadings at around 800 cm^−1^ are characteristic for the Si−H deformation vibration. The bands between 625 cm^−1^ and 580 cm^−1^ are assigned to the Si−O−Si stretching vibrations. The characteristic bands with high loadings reflect variations in the concentration of the silane monomer feed.

This allows the monitoring of the VTMDS feed concentration in-line during the process and, in turn, allows real-time quality control of the extrusion process, helping to make the process faster, more efficient and more cost-effective. It provides a knowledge basis for quantifying the grafting monomer in-line using PLS-R and thus also to quantify the grafting degree.

### 3.9. Determination of Grafting Degree via PLS-R

The in-line Raman data set preprocessed (smoothed, symmetric 21 points, 2nd polynomial order) after the 1st Savitzky−Golay derivation was analyzed using PLS-R. In this analysis, spectral features were extracted from the dataset via PLS-R according to the degree of grafting, given in wt%, from the complex and superimposed information. To this end, the in-line Raman spectra were correlated with the values for the grafting degree as determined by NMR analysis. It was verified that in-line Raman spectroscopy provided an alternative to the costly and time-consuming NMR off-line analysis, thus avoiding the time lag between the process and subsequent analysis of the reaction product. PLS-R effectively reduces the spectral data matrix to a set of orthogonal factors that are predictive of the chemical composition and describes as much as possible of the observed variance in the spectra [17].

**Figure 17:**
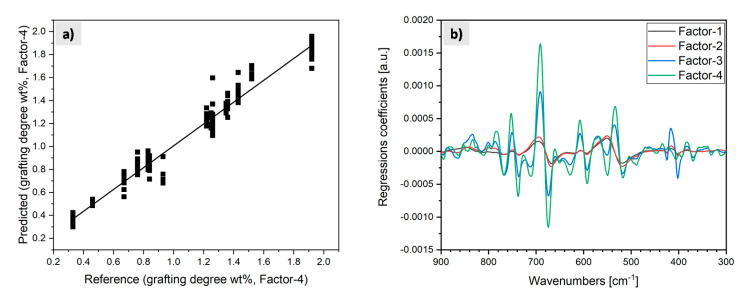
(**a**) Predicted vs. reference values of grafting degree for factor 4 and (**b**) regression coefficients for factors 1–4.

As revealed by comparing the in-line and off-line Raman spectra of the pure substances, the main spectral differences between EPM and EPM-g-VTMDS are found in the wavenumber range between 900 cm^−1^ and 300 cm^−1^. Focusing on these differences, the grafting degree becomes observable in the spectra and consequently this spectral range was used for the PLS−R analysis. The model, calculated with four factors, explained a total of 95% of the data variance. The coefficient of correlation r between the predicted and the reference values in the plot (Figure 17a) is 0.975 and the corresponding Pearson coefficient of determination R^2^ is 0.950, illustrating that the calibration model for the grafting degree wt% is sufficient. The good quality of the PLS-R model is also evident from the root mean square error of calibration (RMSEC), which describes the dispersion of the calibration samples about the regression line and is satisfactorily low (RMSEC = 0.093 wt%). Similarly, the standard error of calibration (SEC), which is RMSEC corrected for bias (i.e., by the mean value over all data points that either lie systematically above or below the regression line), is also very small (SEC = 0.093 wt%). With a difference between RMSEC and SEC of zero, bias is clearly not significant.

The regression coefficients in Figure 17b summarize the relationship between all predictors, the spectral data and the “grafting degree” response in wt%. A large regression coefficient has a large contribution to the modeled response value and hence the corresponding spectral range is important for predicting the grafting degree. In Figure 17b the relative importance of various wavelength ranges to the four model factors can be discerned. It can be observed that all four factors are required to build the model.

The most important regression coefficients for predicting the grafting degree were between 760 cm^−1^ and 680 cm^−1^. In this spectral range, changes in Si−C stretching vibrations are observed. This suggests a positive correlation of the Raman spectra with the grafting degree. A change in the Si−O−Si stretching vibrations is also observed between 610 cm^−1^ and 570 cm^−1^ and is described by the regression coefficients. The effect of variations in the process conditions on the C−C−C framework is evident from the bands around 540 cm^−1^. On this basis, a PLS-R model allowing the prediction of the grafting degree in wt% during the reactive extrusion process was developed. The in-line Raman spectra were regressed versus the known grafting degree in wt%, calculated from the off-line NMR data.

For external model validation, spectra from two material samples with different grafting degrees that were not included in the model building were used and processed with data pre-processing as before in the PLS-R model. The values predicted by the Raman-based model for the grafting degree of these samples were, for the low grafting degree, 0.72 wt% on average, with a standard deviation of ±0.07 wt%. For the high grafting degree, the value was 1.42 wt% ± 0.10 wt%. During the in-line measurements, small variations in grafting degree were observed. For the subsequent off-line ^1^H NMR analysis, these samples were already homogenized by diffusion in the melt. ^1^H NMR spectroscopy yielded grafting degrees of 0.72 wt% for the low-grade grafting and 1.39 wt% for the higher-grade grafting. These values are in good agreement with the ones predicted by the PLS-R model based on the in-line Raman spectra and yield very accurate predictions. Since the off-line NMR data correlate very well with the in-line Raman spectra, it is concluded that the grafting process can be monitored in real-time based on in-line Raman spectroscopy. Thus, the complex and costly NMR spectroscopic off-line quality control with a large time-lag can be substituted by real-time in-line Raman measurements.

## 4. Conclusions

We have presented the synthesis of a novel addition-crosslinkable synthetic rubber based on VTMDS-modified EPM with potential application as a high performance material in various areas. The reactive extrusion process was monitored in-line using a Raman spectroscopic in-situ probe embedded in the extrusion equipment in close proximity to the die tip. It was shown that both the consumption of silane grafting reagent and grafting degree in the EPM-g-VTMDS rubber can be well measured and controlled by process ana-lytical methods in real-time. Process windows for producing this new material with a high grafting efficiency were defined using response surface methodology. The effects and interaction effects of the most relevant process factors were identified and quantified by applying an orthogonal experimental design. It was found that the extrusion process depended in a non-linear and synergistic way on all studied process factors. The VTMDS and DTBPH feed rates were the most important process factors and were involved in synergistic interactions.

## Figures and Tables

**Table 1:** Factors studied and factor levels used in the face-centered experimental design (FCD).

Factor	Name	Unit	Low-Setting (−1)	Centerpoint (0)	High-Setting (+1)
A	VTMDS feed	mol/h	0.1	0.2	0.3
B	DTBPH feed	mol/h	0.003	0.010	0.017
C	temperature				

**Table 2:** Temperature levels employed in the various segments of the extruder to achieve the desired temperature profiles as required by the FCD.

Setting	Temperature Increase	Unit	Segment 1	Segment 2	Segment 3	Segment 4	Segment 5	Segment 6
(−1)	0	°C	80	100	130	160	180	180
(0)	10	°C	100	120	150	180	200	200
(+1)	20	°C	120	140	170	200	220	220

**Table 3:** Experimental data for response surface analysis of the face-centered experimental design with three-level factor settings for the three factors. The experiments are shown in the randomized order in which the single runs were actually performed.

Serial No.	Factor Pattern			Independent Variable			Response Variable	
	A	B	C	A: VTMDS Feed (mol/h)	B: DTBPH Feed (mol/h)	C: Temp. Increase (K)	Grafting Degree (wt%)	Relative Grafting (%)
1	−1	1	−1	0.1	0.017	0	0.67	68.00
2	1	1	−1	0.3	0.017	0	1.43	37.21
3	0	0	0	0.2	0.010	20	1.35	54.50
4	−1	0	0	0.1	0.010	20	0.76	77.27
5	−1	−1	1	0.1	0.003	40	0.46	46.12
6	−1	1	1	0.1	0.017	40	0.84	85.03
7	1	1	1	0.3	0.017	40	1.92	50.05
8	0	0	1	0.2	0.010	40	1.22	49.36
9	0	0	0	0.2	0.010	20	1.36	55.22
10	0	0	0	0.2	0.010	20	1.22	49.31
11	0	1	0	0.2	0.017	20	1.39	56.40
12	1	0	0	0.3	0.010	20	1.52	39.56
13	0	−1	0	0.2	0.003	20	0.72	28.95
14	0	0	0	0.2	0.010	20	1.22	49.56
15	0	0	0	0.2	0.010	20	1.26	50.88
16	0	0	−1	0.2	0.010	0	0.93	37.55
17	1	−1	−1	0.3	0.003	0	0.62	16.14
18	−1	−1	−1	0.1	0.003	0	0.33	33.45
19	0	0	0	0.2	0.010	20	1.25	50.40
20	1	−1	1	0.3	0.003	40	0.83	21.70

**Table 4:** ANOVA for response surface analysis of grafting degree.

Source	Degree of Freedom (df)	Sum of Squares	Mean Square	F-Value	*p*-Value
Model	2.99	6	0.4984	67.65	<0.0001
A-VTMDS feed	1.06	1	1.06	144.26	<0.0001
B-DTBPH feed	1.08	1	1.08	146.93	<0.0001
C-temperature increase	0.1664	1	0.1664	22.59	0.0004
AB	0.174	1	0.174	23.63	0.0003
B²	0.1125	1	0.1125	15.27	0.0018
C²	0.0898	1	0.0898	12.19	0.004
Residual	0.0958	13	0.0074		
Lack of Fit	0.076	8	0.0095	2.41	0.174
Pure Error	0.0197	5	0.0039		
Cor Total	3.09	19	−	−	−

**Table 5:** ANOVA for response surface analysis of grafting efficiency.

Source	Degree of Freedom (df)	Sum of Squares	Mean Square	F-Value	*p*-Value
Model	5275.04	7	753.58	73.34	<0.0001
A-VTMDS feed	2108.59	1	2108.59	205.22	<0.0001
B-DTBPH feed	2259.91	1	2259.91	219.94	<0.0001
C-temperature increase	358.92	1	358.92	34.93	<0.0001
AB	72.24	1	72.24	7.03	0.0211
A²	177.2	1	177.2	17.25	0.0013
B²	163.59	1	163.59	15.92	0.0018
C²	132.17	1	132.17	12.86	0.0037
Residual	123.3	12	10.28		
Lack of Fit	90.43	7	12.92	1.97	0.2372
Pure Error	32.87	5	6.57		
Cor Total	5398.34	19			

**Table 6:** Calibration set for determination of VTMDS and grafting degree via in-line Raman spectroscopy.

Set No.	PCA VTMDS mol/h	Partial Least Squares Regression (PLS-R) Grafting Degree wt%
1	0.1	0.67
2	0.5	1.43
3	0.3	1.35
4	0.1	0.76
5	0.1	0.46
6	0.1	0.84
7	0.3	1.36
8	0.3	1.22
9	0.3	1.39 *
10	0.5	1.52
11	0.3	0.72 *
12	0.3	1.22
13	0.3	1.26
14	0.3	0.93
15	0.1	0.33
16	0.3	1.25
17	0.3	1.26
18	0.5	0.83
19	0.5	1.92

* not used for PLS-R calibration model, only for PLS-R validation.

**Table 7:** Relevant Raman bands for VTMDS, EPM and EPM-g-VTMDS.

Position Raman Band Off-Line (cm^−1^)	Literature Range (cm^−1^)	Molecule Group	Vibration
1597	1615–1590	Si–CH=CH_2_	C=C stretching
1442	1456–1440	−CH_3_ aliphatic	asymmetric
1409	1410–1390	Si–CH=CH_2_	CH_2_ in plane deformation vibration
1305	1305–1295	−(CH_2_)_n_−	CH_2_ deformation vibration
1274	1290–1240	Si−(CH_3_)	Sharp symmetric CH_3_ deformation vibration
1153	1175–1120	C−C−C	C−C−C vibration
1065	1100–1040	C−C−C aliphatic	C−C−C vibration
1039	1100–1040	C−C−C branched	C−C−C vibration
1008	1020–1000	Si–CH=CH_2_	Trans CH wagging vibration
970	973	C−C−C branched	C−C−C stretching
958	980–940	Si–CH=CH_2_	CH_2_ wagging vibration
913	985–800	Si−H	Si−H deformation vibration
846	900–800	C−C−C	C−C−C vibration
839	870–760	Si–CH_3_	Si–CH_3_ rocking vibration
768	765	Si−C	Si−C stretching
737	735–725	−(CH_2_)_3_−	−(CH_2_)_3_− rocking vibration
701	705–670	Si−C	Si−C stretching
678	705–670	Si−C	Si−C stretching
628	624–580	Si−O−Si	Si−O−Si broad symmetric stretching
554	555–530	C−CH_3_/−CH_2_	CH_2_ wagging, C−CH_3_ stretching, CH_2_ rocking
545	625–480	Si−O−Si	Si−O−Si broad symmetric stretching
526	540–485	C−C−C	C−C−C vibration
426	460–420	CH_2_	Wagging CH_2_
303	300	C−C−C	broad C−C−C vibration

## Data Availability

Data are available upon request from the authors.
